# A pre-post trial to examine biological mechanisms of the effects of time-restricted eating on symptoms and quality of life in bipolar disorder

**DOI:** 10.1186/s12888-024-06157-5

**Published:** 2024-10-21

**Authors:** Sheri L. Johnson, Greg Murray, Emily N. C. Manoogian, Liam Mason, J. D. Allen, Michael Berk, Satchidananda Panda, Nandini A. Rajgopal, Jake C. Gibson, Carter D. Bower, Eline F. Berle, Keanan Joyner, Robert Villanueva, Erin E. Michalak, Lance J. Kriegsfeld

**Affiliations:** 1 Department of Psychology, University of California, Berkeley, CA USA; 2 Department of Neuroscience, University of California, Berkeley, CA USA; 3 Centre for Mental Health, Swinburne University, Melbourne, VIC 3122 Australia; 4 Salk Institute for Biological Studies, La Jolla, CA USA; 5 Research Department of Clinical, Educational and Health Psychology, University College London, London, UK; 6 IMPACT Institute, School of Medicine, Deakin University, Geelong, VIC Australia; 7 Department of Psychiatry, University of British Columbia, Vancouver, BC Canada

**Keywords:** Bipolar disorder, Intervention, Time-restricted eating, Sleep, Circadian rhythms, Diurnal, Metabolic health, Quality of life, Depression, Mania

## Abstract

**Background:**

The primary objective of this trial is to examine the mechanisms of time-restricted eating (TRE) as an adjunct to psychiatric care for people with bipolar disorder (BD) with sleep or circadian disruptions. This study builds on prior studies of circadian disruption in BD as well as growing evidence that TRE improves circadian functioning.

**Methods:**

One-hundred fifty participants diagnosed with BD 1 or II will be recruited via advertising in the local community. Main inclusion criteria include: obtaining medical treatment for BD; current sleep or circadian problems; self-reported eating period of ≥ 12 h; no eating disorder or other health conditions that would hinder or limit the safety of following TRE; and not currently experiencing a mood episode, acute suicidality, psychosis, alcohol or substance use disorder. Participants will be asked to complete a baseline period in which daily food intake is logged online for two weeks. After baseline, participants will be asked to follow TRE for 8 weeks and to continue to complete daily food logging during this time. Symptom severity interviews will be conducted by phone or videoconference at baseline, mid-intervention (6 weeks post-baseline), end of intervention (10 weeks post-baseline), and 6 months post-baseline. Self-rated symptom severity and quality of life data will be gathered online at the same time points as symptom severity interviews, and at 16 weeks post-baseline (6 weeks after the TRE period ends). To assess potential mechanisms of change, we will examine the change in diurnal amplitude of ‘clock’ gene expression as a primary mediator at 8 weeks compared to baseline. We will further test whether diurnal amplitude of clock gene expression is predictive above and beyond the role of two covariate potential mediators, glucose tolerance and inflammation at 8 weeks relative to baseline. To provide an index of whether TRE successfully decreases emotional lability, participants will be asked to complete 5 mood assessments per day for 7 days at baseline and at 10 weeks. These mood assessments will be optional.

**Discussion:**

The planned research will provide novel and important information on whether TRE improves sleep/circadian rhythm problems, along with reductions in mood symptoms and improvements in quality of life, for individuals with BD.

**Trial registration:**

ClinicalTrials.gov ID: NCT06555406.

## Background

Bipolar disorder (BD) is a severe, recurrent mood disorder defined primarily by manic symptoms. Different forms of BD have been defined based on the severity and duration of manic symptoms. BD is the fifth leading mental health contributor to global disability [1], and it is all too often tied to unemployment, legal problems, and severe functional impairment [1–5].

Disruptions to sleep/wake cycles and diurnal rhythms in activity and biological functioning are frequently observed in patients with BD and may give rise to, or exacerbate, symptoms. Building on findings over the last decade showing that time-restricted eating (TRE), where eating is limited to 6–10 h during the day, improves diurnal rhythms in metabolic factors and in hormones and the genes underlying circadian function [6–11], the present study seeks to determine if (1) TRE reduces mood symptoms and improves quality of life, and (2) if changes in mood symptoms are related to improved diurnal rhythms in behavior and physiology among those with BD who experience sleep or circadian issues.

### Time-restricted eating (TRE)

The circadian timing system consists of a hierarchy of oscillators controlled by a central pacemaker in the suprachiasmatic nucleus (SCN). The SCN is entrained to the environment via a direct pathway from the retina [12]. Coherence among oscillatory cells in central and peripheral systems is maintained by multi-synaptic, autonomic innervation of peripheral systems by the SCN and central clock control of hormones that target the periphery [13, 14]. Disturbances to this coordinated rhythmic harmony are linked to significant mental and physical health risks [13].

In addition to autonomic innervation and hormonal communication, the timing of food intake can also set the phase of peripheral tissues, without impact on the SCN [15], leading to incongruence between central pacemaker and peripheral systems. Under circumstances where food is ingested during the active phase of the day, systemwide synchrony occurs. However, when eating occurs outside of the active phase, the light-entrained SCN and periphery are at odds in their timing. Seminal work in mouse models established that restricting feeding to the active portion of the day (i.e., the dark phase of the day) improves rhythms in the genes regulating circadian function, improves metabolic functioning (e.g., glucose tolerance and insulin resistance) [7–9, 16–19], and increases longevity independent of changes in caloric intake [20]. Thus, by coordinating peripheral oscillators and the SCN during the day, time-restricted feeding (TRF) helps coordinate circadian rhythms between brain cells in the SCN and cells within other organs (e.g., the liver, lungs, and kidneys) [21, 22]. Indeed, converging evidence across studies indicates that at least some metabolic benefits of TRF result from the resetting the phase of peripheral circadian rhythms [21].

Based on these foundational findings in animal models, the benefits of time-restricted eating (TRE) have received significant attention for human health studies. Across at least 14 randomized controlled trials, substantial metabolic benefits have been observed in glucose regulation and, in many studies, decreased body mass index, including several studies with large sample sizes [23]. In a randomized controlled trial of 80 healthy males, TRE upregulation of the ‘clock’ genes *BMAL1, CLOCK*, and *SIRT1* was observed in serum, suggesting that TRE enhances rhythmic amplitude [24]. As observed under TRF in mice, TRE was shown to shift circadian rhythm of clock gene expression in two smaller studies [10, 11].

High satisfaction and adherence are reported by participants assigned to TRE [21, 25] that likely stem from its health benefits and improvements to sleep duration, sleep efficiency, and reductions in sleep timing variability [25, 26]. Participants report improved energy and restedness within a few weeks of beginning TRE that are sustained for at least six months post-intervention [11, 25]. In fact, several studies report that TRE enhances perceived subjective sleep quality and restedness [11, 25, 27]. Across studies, participants assigned to TRE report significantly improved quality of life ratings from baseline to the 6-month follow-up [27–30]. Of note, TRE does not seem to increase hunger [21, 31], reduce muscle mass [32], or negatively impact cognitive function [33].

### Circadian and sleep disruption in bipolar disorder (BD)

Considerable evidence supports a strong association between biological rhythm disturbances and the pathogenesis of BD [34]. Disturbed sleep-activity rhythms are principal symptoms observed in manic and depressive episodes [35, 36], and these disturbances continue at lower levels for most people with BD during remission [37–40]. Sleep disturbances predict both BD onset and symptom increases in naturalistic studies of sleep, jet lag, activity, and sleep deprivation [40–45]. These issues predict vulnerability to the disorder, with evidence linking (a) BD to single-nucleotide polymorphisms within multiple clock genes, (b) mood variability to polygenic risk scores related to chronotype and low circadian amplitude, and (c) disruptions in sleep/sleep-activity rhythms [46–48] in family members of those diagnosed with BD. Accordingly, there is a need for interventions that target the circadian timing system to stabilize biological rhythms in patients with BD.

#### Biological rhythm intervention in BD

Both in people with BD and in animal models of BD, psychopharmacological and psychotherapeutic treatments impact circadian function, suggesting that improvements to circadian rhythmicity impact treatment outcome. Considerable evidence from animal studies reveals that lithium and antidepressants influence circadian rhythms through actions affecting SCN neuronal firing and clock gene expression [49–53]. In people with BD, differences in neuronal circadian rhythms from patient-derived pluripotent stem cells predict lithium response [54], and psychosocial therapy for mood disorders decreases REM density while improving symptoms [55–57]. Preliminary research shows benefits of pharmacological agents, such as the selective melatonin receptor type I and II agonists ramelteon and agomelatine, as mood stabilizers, suggesting potential therapeutic actions via alterations in circadian rhythms in sleep [58, 59].

Behavioral treatments for BD focus on improving sleep and circadian function, and include interventions such as restricting activity during dark periods [60, 61], interpersonal and social rhythm therapy (IPSRT) [62], and light therapy [63]. However, there are limitations in available treatments designed to address sleep and circadian rhythms in BD and further treatment developments that produce reliable outcomes across individuals and long-term are needed [64].

#### The applicability of the TRE to mood disorders

Although no studies have examined TRE as a BD-specific intervention, some trials considered mood changes among participants without diagnosable mood disorders following TRE. In the only published RCT, TRE decreased depression scores in those with type 2 diabetes compared to a control condition [65]. Likewise, TRE improved quality of life compared to a control condition in a study of shift workers [66]. Significant decreases in pre- to post-depression scores were not observed in uncontrolled studies of TRE [67, 68], potentially due to low depressive symptom levels at baseline.

To examine the potential efficacy of TRE in BD, we completed the first study examining the effects of TRE among patients with BD [69]. 207 participants who self-identified as having BD were surveyed and reported attempting TRE (daily food intake window of < 14 h) for at least several weeks. For all outcomes assessed, 51–66% of participants reported improvements (“better” or “much better”) while applying TRE, and 99% of participants reported gains in at least one domain. These preliminary findings are especially promising given that participants were implementing TRE without the formal support that will be provided in this trial.

### Project aims

The aim of this study is to examine the mechanisms through which TRE influences symptom improvement in BD. We will assign all participants to TRE and will measure diurnal amplitudes and phases of clock gene expression (mRNA) responsible for circadian rhythm generation at the cellular level, as well as dim light melatonin onset (DLMO), the standard measure of circadian phase. We will examine whether changes in the diurnal amplitude predict improved symptoms and quality of life (QoL)—an ideal test of the circadian rhythm hypothesis of BD. To maximize the ability to show that TRE leads to changes in diurnal rhythms, we will recruit individuals who are (a) diagnosed with BD, (b) have at least some symptoms of circadian rhythm sleep–wake disorders or sleep disorders, and (c) report an eating window of ≥ 12 h.

Although our data will be preliminary, we also aim to gather information about two other potential biological mechanisms of TRE: changes in pro-inflammatory cytokines and glucose tolerance. Across studies, TRE rapidly attenuates night-time, fasting, and 24-h glucose levels, with effects sustained across 3–5 weeks of TRE as well as improved glucose tolerance, improved insulin sensitivity and resistance, and lower HbA1c within 3–6 weeks [23]. This is of importance given that over 22% of those with BD show insulin resistance and another 33% have type II diabetes; insulin resistance among those with BD predicts robustly worse symptom course, rapid cycling, treatment resistance to lithium [70], and worse neurocognitive performance. Recent pilot work showed that symptoms of BD were reduced substantially among 50% of patients who were treated with metformin, a diabetes medication [71].

TRE has been found to be associated with significant pre-post reduced pro-inflammatory cytokines, and decreased biomarkers indicative of oxidative stress [72, 73]. Therefore, substantial evidence links inflammation to BD. We will assess and control for changes in pro-inflammatory cytokines and glucose tolerance in examining circadian rhythm normalization as a mediator. This will supply critical mechanistic information to explain individual differences in responsivity to TRE.

Our goal is to examine TRE as an adjunct to psychiatric care for BD. Accordingly, participants will be asked to sustain ongoing medical care for BD.

#### Hypotheses

To examine the mechanisms through which TRE leads to symptom improvements, we will investigate a battery of biological measures at baseline and 8 weeks. We predict that TRE adherence will lead to a change in the amplitudes of diurnal clock gene expression and diurnal phase (DLMO) at 8 weeks, which will lead to a cascade of change in symptoms (mania and depression at week 10, and then a downstream change in QOL at 16 weeks).

- H1: TRE adherence will predict changes in diurnal amplitude of clock gene expression from baseline to week 8.
- H2: The degree of change in diurnal amplitude of clock gene expression (at week 8) will be associated with the degree of improvement in symptoms (week 10), controlling for change in glucose tolerance and inflammation (week 8) and for baseline levels of diurnal amplitude, symptoms, glucose tolerance, and inflammation.
- H3: There will be an indirect effect of TRE adherence on QoL through symptoms.

Ambulatory data will provide opportunity for lagged tests of how biological rhythmicity improvements (actigraphy RA) promote improved sleep, resulting in diminished mood variability (ecological momentary assessment; EMA Mood Zoom) [74].

##### Secondary hypotheses

- H4. TRE adherence will predict improvements in self-rated symptoms of mania and depression at 10 weeks and 6 months post-baseline compared to baseline symptom severity scores.
- H5. The degree of change in sleep and the amplitude of diurnal activity/distal temperature rhythms at week 8 will explain improvement in symptoms (week 10), controlling for baseline symptoms and sleep/diurnal rhythm amplitude.
- H6. The degree of change in metabolic health (CMP) at week 8 will explain improvement in symptoms (week 10), controlling for baseline symptoms and metabolic health.

To provide a more refined index of changes in emotion, we will assess the effects of TRE on daily emotional lability (as assessed using EMA, baseline and post-intervention).

We will conduct exploratory analyses to examine secondary outcomes (e.g., EMA) and potential moderators.

## Methods

This trial will be conducted by an international multidisciplinary team of researchers, clinicians, and lived experience experts. The study was reviewed and approved by the University of California Committee for the Protection of Human Subjects (protocol number CPHS # 2022–10–15725). The trial objectives and protocol align with all aspects of the WHO Trial Registration Data Set (version 1.3.1) and the Standard Protocol Items: Recommendations for Interventional Trials guidelines [75]. We will describe any changes to this trial protocol in publications. We will report scientific findings according to the Consolidated Standards of Reporting Trials (CONSORT) general guidelines and the eHEALTH criteria [76]. Potential financial conflicts of interest will be reviewed annually by the University of California committee for conflicts of interest, and their guidance will be followed in managing any conflicts identified.

### Trial design

The trial is a non-randomized, single arm (TRE) study. The primary endpoint is immediately after the 8-week-long TRE period (end of week 10). The follow-up assessments will be conducted at 6 months after the baseline, supplemented with post-baseline self-reported assessment at week 16. Hypothesized mechanisms of change will be tested at baseline and at 8 weeks post-baseline. The study setting is remote (online food plan materials, questionnaires, and interviews), supplemented with at-home collection of biological data (DLMO, clock genes, actigraphy, CGM) and assays completed by Quest Diagnostics (CMP, glucose tolerance test).

### Inclusion/exclusion criteria

The inclusion/exclusion criteria are designed to maximize participant safety and the applicability of TRE, and secondarily, to maximize the generalizability of the findings. We will recruit adults aged 18–65 years who meet diagnostic criteria for bipolar I disorder or bipolar II disorder per the Diagnostic Interview for Anxiety, Mood, and OCD and Related Neuropsychiatric Disorders (DIAMOND), but not cyclothymia, BD not otherwise specified, or BD due to another medical condition.

To maximize the ability to determine whether TRE improves sleep- or circadian-related outcomes, the entrance criteria will include at least some current sleep or circadian sleep–wake concerns. We do not expect this to greatly diminish generalizability, as more than 75% of those with BD report sleep or circadian concerns [77]. This inclusion criteria will be met by any one of the following:

- Score of ≥ 8 reflective of subsyndromal insomnia on the Insomnia Severity Index (ISI; [78–80])
- Early chronotype (midpoint of sleep on free days < 4.16 h) or late chronotype (midpoint of sleep on free days > 4.53) on the MCTQ
- Disrupted social activity rhythms, as indicated by a score ≥ 30 on the Brief Social Rhythm Scale (BSRS) [81]
- Sleep (insomnia, hypersomnolence) or circadian sleep–wake (delayed phase, advanced phase, irregular sleep–wake, non-24-h sleep–wake-type) disorder diagnoses on the Structured Clinical Interview for Sleep Disorders-Revised (SCISD-R; [82]).

Other inclusion criteria include the following:

- English language adequate for completion of questionnaires or interviews, as indicated by speaking English for at least 10 years, speaking English at home, or proficiency as observed during the screening interview.
- Receiving medical care for BD (referrals will be provided for those who would like to begin care)
- Mood-stabilizing medication regimens need to be stable for at least one month. Those with BD II per the DIAMOND will be permitted to engage in the trial if not taking mood-stabilizing medication, as long as their provider has supported this treatment plan and continues to see them at least once every 3 months.
- Currently eating ≥ 12 h per day at least twice per week
- Able to operate the camera function and respond to surveys by smartphone (loaner phones will be provided as needed)

Exclusion criteria include the following:

- Current episode of depression, hypomania or mania, or psychosis (per the DIAMOND).
- Suicidal ideation coupled with current plan or intent or history of attempt as assessed by the Columbia Suicide Severity Rating Scale (C-SSRS).
- Eating disorder diagnosis (self-reported history of treatment or diagnosis, Short Eating Disorder Examination Questionnaire (EDE-QS; [83]) scores above clinical concern thresholds for eating disorders (≥ 15) or DIAMOND diagnosis of eating disorder (anorexia, bulimia, binge eating disorder or other specified eating or feeding disorders).
- Past 3-month alcohol use disorder or substance use disorder (per the DIAMOND)
- Current shift work or responsibilities, such as providing care, that would chronically disrupt sleep (i.e., > 3 h between 22:00 and 05:00 h for at least 1 day/week)
-  > 5 kg weight change in the past 3 months
- Conditions that would interfere with ability to take part in TRE, including type I diabetes, pregnancy, breastfeeding, uncorrected hypo- or hyperthyroidism, or gastrointestinal conditions impairing nutrient absorption.
- Medical conditions that could confound immune or other study measures, such as HIV, AIDS, multiple sclerosis or lupus
- Medications contraindicated for fasting; clozapine; glucose-lowering medications; diabetes or obesity-related injections; medications requiring food early in the morning or late in the evening, corticosteroids; GLP-1 agonists (such as Semaglutide) will not be an exclusion criteria if weight is stabilized
- Cognitive deficits as noted during the initial interview or as indicated by low performance on the Brief version of the Orientation Memory Concentration Test (Weighted, reverse score < 20)
- Failure to complete screening and baseline questionnaires adequately (see strategies to maximize data quality procedures below).
- Failure to complete 7 days of dietary logs adequately (e.g., at least 2 entries per day, covering at least a 5-h eating window) during the baseline period.

### Recruitment and assessment

Recruitment and screening procedures will be parallel with those reported previously [69]. Briefly, participants will be recruited from community and clinic centers, and we will advertise to oversample those in the early stage of their illness (within 5 years of onset). Screening will begin with completion of online informed consent, and then an online screening survey to assess medical and demographic criteria, and to complete self-rated psychiatric screeners for eating disorder symptoms, alcohol, substance and psychosis symptoms. Those who meet criteria within the screening survey will be scheduled for a remote interview consisting of the DIAMOND interview to assess psychiatric diagnoses, and the Structured Clinical Interview for Sleep Disorders-Revised (SCISD-R) [82] to assess sleep disorders (insomnia, hypersomnolence disorder) and circadian sleep–wake disorders (irregular sleep–wake disorder and non-24-h sleep–wake type disorder). Participants will be paid $25 USD per hour for baseline and follow-up assessments but not be paid for other components of the study (screening, coaching and time spent implementing the dietary plan).

#### Screening interview

Participants who do not have a medical provider for their BD or who are experiencing acute mood episodes or suicidality will be provided with support and referral information and invited to recontact us once treatment and symptom stability are in place, with follow-up planned. Similarly, those who are not weight stable will be invited to recontact us once their weight stabilizes.

#### Baseline assessment

Participants who meet the eligibility criteria will be asked to complete a two-week baseline. The baseline assessments will include daily food logs, sleep diaries, and EMA probes sent via smartphone app notifications, interview measures, self-report questionnaires, actigraphy, and biological measures.

##### Food logs

Throughout the baseline period, participants will be asked to complete daily online food logs to monitor food intake and timing. The logs are designed to be simple to use and to lower burden by allowing participants to take a photo of their food or to briefly note the food without the burdens of inputting portion size or calories. Participants can complete their diet log in real time, although missed entries can be added retroactively. The validity of this daily logging approach is well-validated [84].

Continuing with the study will be contingent on adequate completion of food logs for at least 7 days during the baseline period. Adequate logging on a given day will be defined as entry of at minimum two logs at least five hours apart.

Strategies to foster logging adherence are detailed elsewhere [69]. At the end of the first week, participants with no days of adequate logging will be excluded from the study (unless we identify a barrier that can be addressed). After 10 days, those who have completed less than 50% of their assigned logs will be reminded that study continuation will depend on successful logging and given an opportunity to receive support around barriers to logging; if warranted (e.g., the patient experienced technical or travel barriers), we will extend the baseline period for four additional days.

##### EMA assessments

*Optional.* Starting on day 5 of the baseline period, for individuals who have opted into the EMA portion of the study, we will send probes by app to gather sleep and affect measures. Participants will be asked to complete the Core Consensus Sleep Diary (Core CSD) [85] each morning for 10 days. They will also be asked to complete 5 EMA prompts per day for 7 days to assess mood fluctuations (detailed below). We will send the Core CSD and EMA probes on at least 2 free days for each person (e.g., one Saturday and Sunday).

##### Interview and self-report assessments

After at least 3 days of adequate logging, participants will be sent links to schedule the baseline interview; Montgomery-Asberg Depression Rating Scale [MADRS]) [86]; Young Mania Rating Scale [YMRS]; [87]; and to complete baseline questionnaires. Baseline questionnaires will include demographic information, symptoms, sleep, and functional information (Patient Mania Questionnaire [PMQ-9]; [88]), Patient Health Questionnaire [PHQ-9]; [89], and Brief Quality of Life in Bipolar Disorder (QoL.BD; [90]), PROMIS Sleep Disturbance and Sleep-Related Impairment [91], Sleep Household Environment and In-Bed Behaviors [92], and World Health Organization Disability Assessment Schedule (WHODAS 2.0-Brief; [93]). The participants will also be asked to complete measures of potential moderators (Household Food Security Survey [HFSSM]; [94]), Positive Urgency [95], and for women in appropriate age ranges, the Menopause-Specific QoL Sleep [hot-flash] items [96, 97]).

##### Actigraphy and biological measures

For 10 days during baseline, participants will be asked to wear the Dexcom G6 continuous glucose monitor to provide 24-h continuous glucose levels, the GENEActiv wristband (Activinsights Inc.) to collect activity and sleep data, and a wrist-worn iButton to collect distal temperature. Participants will perform at home salivary collection for DLMO and inflammation assessment. They will be asked to collect 6 buccal cheek swabs (every 3 h) across the course of one day to assess peripheral clock gene expression. Participants will be asked to visit Quest Diagnostics for a comprehensive metabolic panel (CMP) and glucose tolerance test.

#### Assessments after the baseline period

Participants will be asked to continue daily food logging throughout the 8-week intervention period. At 6 months after the end of baseline, participants will log their food (first intake and last intake) for 10 days in the app.

##### Mid-intervention

Participants will be asked to complete 8 days of sleep diaries (core CSD). Participants will wear the Dexcom G6 CGM to collect glucose levels continuously for 2 weeks and wear the GENEActiv (Activinsights Inc.) wristband to collect activity/sleep data for 3 weeks. Participants will perform at home salivary collection for DLMO assessment and collect buccal cheek swabs to assess peripheral clock gene expression. Participants will visit Quest Diagnostics for a CMP and glucose tolerance test.

##### Symptom, mood, and quality of life (QoL) assessments

Symptom severity and LIFE interview assessments will be conducted at the midpoint, at the end of the 8-week TRE period (week 10) and at 6 months after the end of baseline. Self-rated assessments of symptom severity and sleep/circadian variables will be conducted at each of those assessments and at 16 weeks post baseline. The EDE-QS will be administered at midpoint and post intervention. Participants will be asked to complete EMA for 7 days post intervention. Secondary outcome measures will be assessed post intervention.

#### Training and reliability

Before commencing study interviews, interviewers will be trained to standardize administration and to attain high interrater reliability on all observer-rated measures (the DIAMOND, SCISD-R, C-SSRS, LIFE, MADRS and YMRS). Interrater reliability reviews will be conducted monthly on randomly selected recordings throughout the trial. If rater drift is identified, assessors will be retrained.

### Strategies to maximize data quality

As described elsewhere [69], we will use multiple criteria to assess potentially careless responses. These include strategies to detect and respond to inattentive responding, and data cleaning techniques.

### Risk management

As detailed elsewhere [69], risk management procedures are based on online intervention and psychoeducation websites for BD [98, 99]. These will include obtaining a release to contact a primary provider, careful training of all interviewers and coaches in how to assess and respond to high-risk symptoms and suicidality, routine assessments of possible relapse and emergent symptoms, and guidelines for providing sensitive referrals based on the urgency of the situation.

#### Adverse events

In addition to the risk management procedures, we will take several steps to minimize the potential for adverse events. For participants who experience the onset of symptoms, we will consider with participants whether it would be helpful to take a break from the study if requirements might interfere with placing a focus on recovery. For participants who choose to take a break from the study, and interviewers will schedule a time to check in to consider restarting trial engagement. Techniques for routinely assessing adverse events, responding to signs of adverse events, and reporting such events are detailed elsewhere [69].

#### Confidentiality

To protect confidentiality, we will hold to privacy standards that match or exceed those of all countries that we are recruiting in. For US participants, we have obtained an NIH Certificate of Confidentiality. All study personnel will be trained in confidentiality and privacy, through online CITI training as well as direct training provided by members of the executive team.

All participants will be assigned a study ID to be used in place of the participant’s name on all assessment materials. We will destroy the list linking IDs and names after study completion. Data for this project will be gathered using REDCap, a highly secure web-based research data collection system. REDCap provides several features to protect data security, including secure passwords required for staff log-in, well-defined levels of data access based on a staff member’s role in the project, data storage on servers located in ITG’s Advanced Computing Center which has locked physical security; use of an OHSU firewall and a second ACC firewall; and data transmissions that are encrypted with industry-standard SSL methods. Video contact with participants will be conducted by secure, HIPAA-protected zoom calls; participants have the option to turn off their camera during those meetings or to request telephone interviews in place of video calls.

### TRE content

We will be careful to inform participants that our study does not involve dieting and that we expect them to sustain their caloric intake throughout the time following the TRE food plan. TRE will be administered as self-help via online instruction supplemented with optional coaching sessions and participant logging as previously described [69].

To minimize attrition and nonadherence, the food plan period is limited to 8 weeks. Participants are welcome to continue to access food plan materials and follow the food plan after the assigned 8 weeks. Individuals with personal and family experience with BD reviewed intervention content and their suggestions about the usefulness and modification of the material were incorporated [100, 101].

Because previous research has shown that interactive support improves adherence to web interventions [102], participants will be provided with opportunities to schedule 15-min online coaching sessions or to email questions about implementing TRE. We will encourage an initial coaching session. At one, three, five, and seven weeks into the TRE period, they will receive a link offering a chance to contact us or a link to a calendar to schedule a coaching appointment. Participants will be instructed that the scope of this coaching is narrow and will only focus on TRE. Participants will be invited to bring significant others, family members or household members to the coaching sessions if they’d like. Participants can send as many messages as they like using a contact form but will receive only one response per week. Coaching sessions will also be limited to no more than once a week.

Coaches will receive guidelines and will meet with licensed clinical psychologists regularly throughout the intervention for support and to ensure that their coaching maintains fidelity to TRE and the dietary changes. Coaches will aim to provide a supportive, nonjudgmental environment where participants can discuss barriers and difficulties in implementing dietary plans and can receive behavioral tips. The focus of coaching will be on collaborative problem solving and empowerment in addressing barriers. Coaches will aim to help participants lessen self-criticism, to avoid overly ambitious or restrictive dietary changes, and to be sensitive to mood fluctuations that may make following this type of program difficult. Coaches will aim to help participants identify material in blogs, video clips, or other barriers materials that might help address the concerns raised.

Participants will have access to a website of information designed to address barriers to following their food plan (e.g., coordinating timing of food with family meals or holidays), improving sleep, or addressing light exposure. Barriers were identified by reviewing previous studies using TRE, by conducting a survey of individuals with BD who had tried TRE, and by reviewing concerns raised by those with lived experience of BD and/or following dietary programs. For each major barrier, brief tips are available, which vary in format (written, audio, or video); all tips are designed to take 2 min or less. To help participants access relevant materials, they will be sent a quick survey at the end of weeks 3, 5, 7, and 9 in the intervention period or post baseline asking if they had experienced any barriers and providing the option to view tips relevant to the barriers endorsed.

### Measures

**Fig. 1:**
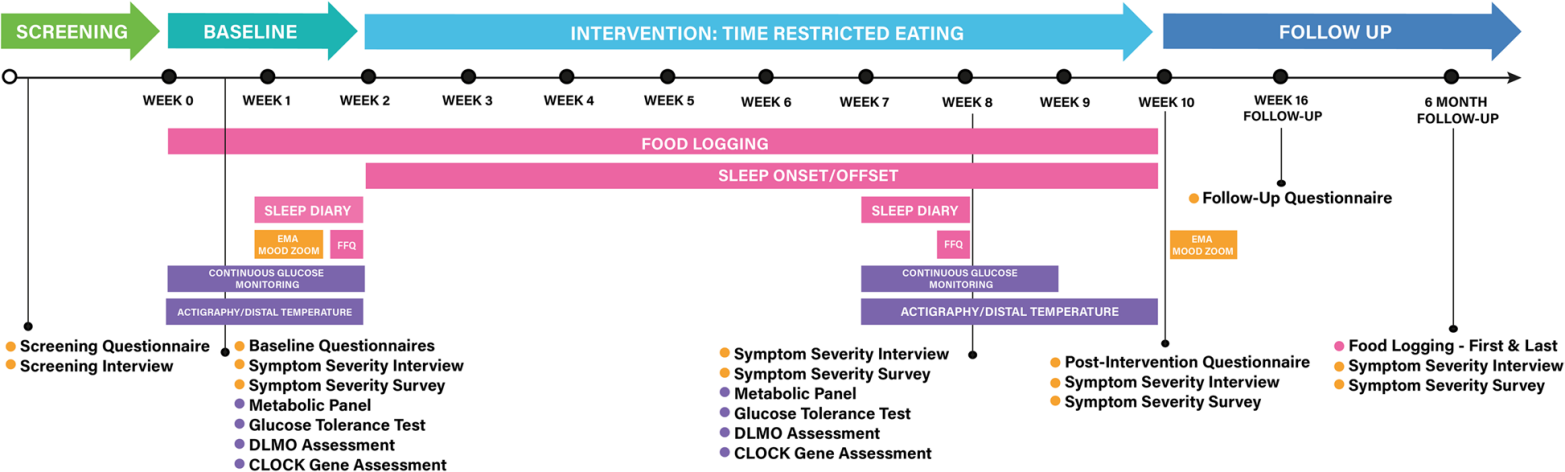
Assessment timing

The schedule of the assessments is provided in Fig. 1 and Table 1. Assessments will be performed as closely as possible to specified time points. Participants will be sent calendar links to sign up for their interview assessments. Self-report assessments will be completed online via a secure, encrypted online survey platform (REDCap). Probes for logs will be sent by text message for completion via cellphone or computer, and EMA probes will be sent via a smartphone app.

**Table 1:** Schedule of assessments

Measure	Format	Screening	Baseline	Mid-Intervention (Week 8)	Post-Intervention (Week 10)	Week 16 Follow Up	6 Month Follow Up
***Screening Self-Report***							
DIAMOND Self Report Screener Psychosis Subscale	SR	X					
Alcohol Use Disorders Identification Test (AUDIT)	SR	X					
Drug Use Disorders Identification Test (DUDIT)	SR	X					
Short Eating Disorder Examination Questionnaire (EDE-QS)	SR	X		X	X		
Insomnia Severity Index (ISI)	SR	X					
Munich ChronoType Questionnaire (MCTQ)	SR	X					
Brief Social Rhythms Scale (BSRS)	SR	X					
***Screening Interview***							
Orientation, Memory and Concentration Test (OMC)	I	X					
Diagnostic Interview for Anxiety, Mood, and OCD and Related Neuropsychiatric Disorders (DIAMOND)	I	X					
Columbia Suicide Severity Rating Scale (C-SSRS)	I	X					
Structured Clinical Interview for Sleep Disorders—Revised (SCISD-R)	I	X					
***Primary Outcome Measures***							
Montgomery-Asberg Depression Rating Scale (MADRS)	I		X	X	X		X
Young Mania Rating Scale (YMRS)	I		X	X	X		X
Brief Quality of Life in Bipolar Disorder (QoL.BD)	SR		X	X	X	X	X
***Secondary Outcome Measures***							
Longitudinal Interval Follow-up Evaluation (LIFE)	I			X	X		X
Patient Mania Questionnaire (PMQ-9)	SR		X	X	X	X	X
Patient Health Questionnaire (PHQ-9)	SR		X	X	X	X	X
Patient Global Impression (PGI)	SR			X	X	X	X
Clinician Global Impression (CGI)	I			X	X		X
*Optional:* EMA of Emotion States: Mood Zoom (MZ)	App		7 days		7 days		
WHO Disability Assessment Schedule 2.0-Brief	SR		X		X		
General Anxiety Disorder-7 (GAD-7)	SR		X		X		
Rapid Measurement Toolkit-20 (RMT20) panic, social anxiety and PTSD subscales	SR		X		X		
Godin Shephard Leisure-Time Physical Activity Index	SR		X		X		
***Intervention Acceptability Probes***							
Ratings of the acceptability of the Intervention	SR				X		
***Adherence***							
Food Logging	SR		Daily	Daily			10 days- first & last intake
Sleep Household Environment and In-Bed Behaviors	SR		X		X		
***Potential Mechanisms of Change***							
Continuous glucose monitoring	HC		2 weeks	2 weeks starting week 7			
Actigraphy/Distal Temperature	HC		2 weeks	3 weeks starting week 7			
Metabolic Panel	QD		X	X			
Glucose Tolerance Test	QD		X	X			
DLMO	HC		X	X			
Clock Genes	HC		X	X			
Inflammation	HC		X	X			
Core Consensus Sleep Diary (CORE CSD)	SR		10 days	10 days			
PROMIS: Sleep Disturbance and Sleep-Related Impairment Scales	SR		X	X	X		X
***Potential Moderators***							
Positive Urgency Scale	SR		X		X		
Household Food Security Survey (HFSSM)	SR		X				
Menopause QoL items on sleep disruption (for women ages 45–65 (MENQoL)	SR		X				
Food Frequency Questionnaire (FFQ)	SR		3 days	3 days			
Somatotherapy	SR	X	X	X	X	X	X
Demographics	SR		X				
Credibility and Expectancy Questionnaire – Credibility Set	SR		X				

*App* Application, *I* Interview, *SR* Self-report, *HC* Home collection, *QD* Quest Diagnostics

#### Screening questionnaires

All screening questionnaires are well-validated, commonly used indices.

##### DIAMOND Self Report Screener psychosis subscale [103]

We will administer the DIAMOND Self Report Screener psychosis subscale to assess psychotic symptoms. For individuals who endorse at least one symptom, we will administer the DIAMOND psychosis module to further evaluate participant eligibility.

##### Alcohol Use Disorder Identification Test (AUDIT; [104–106])

We will administer the AUDIT to screen for alcohol use symptoms. For individuals who score above the threshold on the AUDIT (≥ 8), we will administer the DIAMOND alcohol use disorder module to further evaluate participant eligibility.

##### Drug Use Disorder Identification Test (DUDIT; [107, 108])

We will administer the DUDIT to screen for drug use symptoms. For individuals who score above the threshold on the DUDIT (≥ 6 for men; ≥ 2 for women and nonbinary individuals), we will administer the DIAMOND substance use disorder module to further evaluate participant eligibility.

##### Insomnia Severity Index (ISI; [78, 80])

The ISI is a 7-item index of insomnia symptoms, including sleep disturbance and daytime consequences of poor sleep.

##### Short Eating Disorder Examination Questionnaire (EDE-QS; [83])

The EDE-Q short form is a 12-item self-report measure designed to assess a range of eating-related symptoms, including restraint, eating concern, shape concern and weight concern [109]. The EDE-QS has shown strong internal consistency (Cronbach’s α = .913, high temporal stability (ICC = .93), and high correlations with eating disorder diagnoses and original EDE-Q scores (r’s > 0.81).

##### Munich Chronotype Questionnaire (MCTQ; [110])

The MCTQ is designed to assess chronotype, with a focus on sleep timing during free days.

##### Brief Social Rhythms Scale (BSRS; [111])

The BSRS assesses the irregularity of engagement in daily activities such as mealtimes and social interaction on workdays and free days, and has been validated in BD.

#### Screening interviews

Our chosen Interviews have all been shown to have strong inter-rater reliability and validity.

##### Orientation Memory Concentration test (OMC; [112])

The OMC is a brief test of mental status.

##### Diagnostic Interview for Anxiety, Mood, and OCD and Related Neuropsychiatric Disorders (DIAMOND [103])

The DIAMOND is a well-validated, semi-structured diagnostic interview [103]. We will use the DIAMOND to confirm bipolar I or bipolar II disorder diagnoses, to assess episodes of major depressive disorder and diagnostic exclusion criteria (eating disorders, including symptoms consistent with other specified eating or feeding disorders; and for those with scores above the threshold on the AUDIT, DUDIT, or psychosis screener, the presence of alcohol use disorder, substance use disorder, or psychosis).

##### Columbia Suicide Severity Rating Scale screening version (C-SSRS; [113])

The C-SSRS is a brief, commonly used, semi-structured interview designed to cover suicidal ideation and behavior. We will use the C-SSRS to assess our active suicidality exclusion criteria.

##### Structured Clinical Interview for Sleep Disorders—Revised (SCISD-R; [82])

The SCISD-R is a semi-structured interview designed to diagnose common sleep disorders. We will use the SCISD-R to assess insomnia, hypersomnolence disorder and circadian sleep–wake disorders.

#### Primary outcomes

Our primary outcome measures will be: interview-based assessments of mood symptoms, and a brief self-report measure of Quality of Life, which were chosen based on strong psychometric characteristics, including high interrater reliability for the interview-based measures. Where possible, the symptom severity interviews will be conducted by raters who are unaware of the participant’s intervention status (which is feasible as our team is conducting a parallel study involving the Mediterranean diet). Where a rater becomes inadvertently aware of the intervention status, we will attempt to replace the interviewer for that participant.

##### Montgomery-Asberg Depression Rating Scale (MADRS; [114])

The MADRS is a brief measure designed to assess the severity of depressive symptoms.

##### Young Mania Rating Scale (YMRS; [87])

The YMRS is an 11-item interview designed to assess the severity of manic symptoms.

##### Quality of life

We will use the Brief Quality of Life in Bipolar Disorder (QoL.BD) questionnaire [90], which was designed to capture key life domains for individuals with BD and to be pragmatic for repeated assessments. The measure shows strong internal consistency and correlations with objective measures of functioning [109]. The brief short version is based on factor-analytic methods, has been validated for online use, and has been shown to be sensitive to change with intervention [115, 116].

#### Secondary outcome measures

To allow for more frequent and affordable assessments, we will supplement our core interviews with the Wellcome Trust recommended self-report measures for depression and mania: the Patient Health Questionnaire (PMQ-9 for mania and PHQ-9 for depression); [88, 89]. Both are brief scales that have been shown to have high internal consistency and to be sensitive to intervention effects. For comparison with the findings of other studies, we will also gather the Clinical Global Impression (CGI) and Patient Global Impression (PGI) indices.

##### Longitudinal Interval Follow-Up Evaluation (LIFE; [117, 118])

To provide more coverage of mood variability between assessment windows, we will also gather LIFE scores. We include the LIFE to enrich our ability to “catch” changes between follow-ups [111].

##### EMA of emotion states

In an optional add-on to the study, we will examine the temporal dynamics of mood with ecological momentary assessment (EMA) assessments. EMA has the advantage of providing a potentially more sensitive indicator of shifts in mood variability [119] that is less affected by recall bias. At baseline and again immediately after intervention, participants will be sent 5 surveys per day on their smartphones. Surveys will be randomly spaced, on average, 135 min apart to allow for three hours between the final survey of the day and their usual bedtime. Participants will have up to 80 min to respond to each survey and will receive reminders if they do not respond within the first 40 min.

In our EMA, we will use the Mood Zoom probes, which have been validated in BD [120]. Our core index derived from dimension reduction of the emotion probes is the mean variability in daily negative affect (mean square of successive difference). As a secondary test of proposed mechanisms, we will also assess whether TRE, as a circadian intervention, leads to decreased evening levels of arousal.

##### Broader clinical outcomes

To harmonize our findings with those of other Wellcome Trust grants, we included three measures as secondary outcome measures: the World Health Organization Disability Assessment Schedule 2.0—Brief (WHODAS 2.0-Brief; [93])**,** GAD-7 [121] and the panic, social anxiety and PTSD subscales of the Rapid Measurement Toolkit (RMT20; [122]). The WHODAS 2.0-Brief is a 12-item survey that assesses overall role functioning across major life domains. The GAD-7 is a commonly used measure of generalized anxiety symptoms. The RMT20 is a brief screening tool developed using IRT analyses and well validated against clinical diagnoses [122].

#### Potential mechanisms of change

As shown in Table 1, we will gather measures of potential change mechanisms at baseline and mid TRE. Our core hypotheses focus on diurnal clock gene amplitude, but we will gather a multi-modal battery of indices of circadian function (Fig. 1).

##### Diurnal clock gene assessment

Clock gene expression from oral cheek swabs will be used to assess peripheral circadian physiology at 8 weeks. Participants will be asked not to eat, drink, chew gum, or smoke within 60 min of each sample collection, and to rinse their mouth with water prior to collection. Participants will be provided with a ‘kit’ containing collection tubes in a foam rack, sterile buccal swabs, a cooler with blue ice packs, and an instruction sheet with a checklist. Participants will be asked to collect samples every 3 h, beginning 1 h after habitual wake time, for a total of 6 samples. Buccal mucosa samples will be collected by participants gently swabbing the inner cheek on both sides using a cytological brush (i.e., a buccal swab). After collection, participants will immediately place the oral mucosa samples into RNAlater reagent (Sigma-Aldrich, St. Louis, MO, USA) already contained in collection tubes. The cooler with samples will be collected by an experimenter from the participants’ homes and measured in the lab with real-time quantitative polymerase chain reaction.

##### Sleep and diurnal rhythm assessment via actigraphy and distal temperature

We will use the GENEActiv (Activinsights Inc.) wristband to record continuous activity counts and ambient light-exposure. Actigraphy data will be collected during baseline for two weeks and for 3 weeks at week 7. An integrated RGB light sensor records light measurements to help assess the impact of ambient light on human circadian and sleep physiology. The GENEActiv wristband is capable of a month of continuous recording and will not need to be recharged during the period of wear. The device is waterproof, allowing participants more freedom to wear the watch when completing normal activities. Actigraphy data are collected via an onboard 3-axis accelerometer and will be analyzed for sleep onset, offset, duration, and efficiency. Diurnal rhythm amplitude and stability will also be assessed using this activity data. Participants will be provided instructions and will not have access to data as it is collected on the device. Clocklab software (Actimetrics, Lafayette IN) will be used to calculate diurnal amplitude, mesor, and acrophase. Interdaily stability (IS), and intradaily variability (IV) will be calculated using non-parametric analyses in Clocklab. Sleep onset, offset, duration, and efficiency indices will be determined using validated algorithms from Activinights.

As a secondary measure of diurnal rhythmicity, we will use iButtons to record wrist temperature (temperature logged every 5 min). The iButton is a small (17.35 mm diameter × 5.89 mm depth) data logger that can be used to record human skin temperature at selected intervals and is regularly applied to studies of circadian rhythms and sleep/wake research (e.g., [123, 124]). iButtons will be used to chronically record wrist skin temperature when actigraphy measures are collected. The iButtons will be placed on the top of the wrist and secured in place with self-adherent cohesive wrap tape made of medical-grade non-woven, hypoallergenic fabric.

##### Dim Light Melatonin Onset (DLMO)

Prior to collection, a COVID screening questionnaire (see attachments) will be administered. Sample collection will not commence if any response is in the affirmative. To assess central circadian timing, DLMO will be examined during the baseline period and at week 8. While historically conducted in a laboratory setting, home collection of DLMO has been validated (e.g., [125, 126]). Prior to collection, an experimenter will perform a home visit to determine the best location to collect samples based on ambient lighting. If needed, blackout curtains will be provided. To ensure dim lighting requirements are adhered to, participants will use the MiEye Wearable Light Sensor (Circadian Health Innovations, Australia). A demonstration of how to collect a saliva sample will be given, and participants will receive a home saliva collection kit. Participants will be instructed not to consume any alcohol or caffeine at least 24 h before each home phase assessment. Participants will be instructed to prepare food ahead of time so they can eat between the hourly saliva samples at home if desired.

The home saliva collection kit consists of an insulated cooler with removable ice pack, a paper checklist with space to report any atypical events, a tube rack, collection tubes, and a soft toothbrush. Participants will also be offered battery-powered tea candles to assist in ambient lighting in the collection area, bathrooms, and any other rooms in which visits might occur during collection. Participants will be instructed to follow the checklist whenever alarms sound on the timer, and to check off events when completed.

A phone alarm will occur 30 min before the first saliva sample to remind participants to ensure provided blackout curtains are placed. Participants are asked to brush their teeth with the provided toothbrush if they have eaten any food, rinse with water, and remain seated until after the next saliva sample. At the time when a saliva sample is to be collected, the checklist prompts participants to open the first labeled collection tube and use a passive drool approach to collect around 1 ml of saliva. Participants will collect samples hourly beginning 5 h before their habitual bedtime until 1 h after their habitual bedtime. Samples will be placed into the freezer at the end of collection and samples and kits will be collected at a time scheduled by the participant.

To supplement our primary indices of circadian function, we will examine changes in sleep (the gold standard CORE CSD measures of quality of sleep and reduced variability in sleep midpoint, along with PROMIS sleep disturbance and sleep-related impairment, and actigraphy and temperature measures). To assess whether the sleep and light guidance offered as part of the TRE intervention leads to improved sleep hygiene, we will gather the Sleep Household Environment and In-Bed Behaviors [92].

#### Secondary mechanism of change measures

##### Pro-inflammatory cytokines

To examine inflammation, participants will collect a morning (1 h after habitual wake time) and evening (12 h after first sample collection) salivary sample to assay for interleukin-1 and -6.

##### Continuous glucose monitoring

Continuous glucose monitoring will be conducted to examine daily patterns of glucose activity and their amplitude. Continuous glucose monitoring will be performed using the Dexcom, G6 CGM. Researchers will place the CGM onto the back of the upper arm of participants to ensure proper placement which will also activate the CGM. Participants will not have access to data while wearing the monitor. Participants will wear the G6 CGM monitor for 10 days at both baseline and week 6, before removing the sensor using instructions provided.

##### Comprehensive metabolic screen and glucose tolerance test

A broad metabolic screen and glucose tolerance test will be conducted by Quest Diagnostics in a single visit to measure metabolic health parameters at both baseline and week 8. Participants will log into a Quest Diagnostics portal specific to this study to schedule the appointment.

#### Intervention acceptability probes

We will use acceptability probes previously validated in the ORBIT clinical trial for BD [98], rated on a scale from “Strongly Disagree” to “Strongly Agree”. Ratings of “agree” or “strongly agree” for each item will be considered acceptable. Our primary index of acceptability will be the percentage of individuals who endorse that they agree or strongly agree that they would recommend the food plan to a friend, but we will share responses on each item for transparency.

We will supplement these probes with questions to assess whether individuals perceive that their mania, depression, sleep, or quality of life improved or worsened with TRE. As a further check of potential negative outcomes, participants will be asked open-ended queries regarding problems associated with TRE.

#### TRE adherence

We will score adherence using well-validated metrics to evaluate the time of first and last caloric intake each day. To reduce skew from infrequent deviations from the eating plan, we will define the time interval that contains 95% of intake events [127]. Consistent with other US and European studies of TRE, we will define adequate logging for a given day as entry of at least two intake events covering at least a 5-h window. Focusing on the days with adequate logs, we will calculate the percentage of the days in which individuals met the eating window goal. High adherence will be defined as meeting this standard on at least 78% of the days with adequate logs. As supplemental data, we will report the percentage of days logged and the percentage of days in which individuals logged adequately and followed the planned window.

#### Potential moderators

We will gather several variables to assess their role in moderating outcomes. These include data on the types of psychotropic and sleep medicines, doses, and adherence levels at each self-report assessment to calculate summary scores for each major receptor type. At baseline, we will gather data on the Positive Urgency Scale [128], food insecurity (HFSSM; [94, 129]), and Menopause QoL sleep items [96, 97], as potential predictors of intervention outcome. At baseline and at 6 weeks, we will gather a Food Frequency Questionnaire to assess inadvertent changes in dietary intake (e.g., less dessert consumption after participants stop midnight snacks).

### Reasons for discontinuation from study

To support intent-to-treat analyses, we will attempt to gather final outcome assessments for all participants who discontinue the study (except those who explicitly discontinued or were withdrawn on ethical grounds. This final outcome assessment will include an intervention acceptability survey, self-rated mania, depression, and QoL; and, if participants are willing, the YMRS, MADRS, and the LIFE. The trial coordinator will attempt to contact participants who withdraw by phone to ask the main reasons for discontinuation. Should adverse events be discovered, those will be reported.

### Statistical analysis plan

Before conducting analyses of hypotheses, we will examine whether values appear to be missing at random. If this missing value imputation assumption is supported, we will impute data using 20 resamples, using demographic (sex/gender, age), baseline symptom severity, and symptom history variables (number of episodes) to predict missing scores. For comparison, we will perform analyses without imputed values. Data cleaning will be completed as described above in data quality. Statistical analyses will be conducted in accordance with the International Conference on Harmonization E9 statistical principles.

Before conducting analyses of hypotheses, we will carefully examine potential confounds. We will avoid adding any covariates that were entangled with our core mechanisms (e.g., sleep and circadian processes). With this in mind, we will only consider covariates for inclusion in models if they could be confounding our main effects– that is, those that are significantly correlated with both our IV (treatment adherence) and DVs (symptoms and QoL). Power analyses were based on inclusion of two covariates, and we would hope not to exceed this number of covariates. If more than two potential covariates are identified that appear significantly and uniquely correlated with both the IV and DVs, we would redo our power analyses, but at this point, we would be able to consider the covariance matrices, allowing for more careful estimation of true statistical power. We would consider these issues without generating the models of how our treatment variables actually influence symptoms and QoL, so as to avoid p-hacking or “fishing”. Only if those power analyses suggested that we could include more than 2 variables as confounds would we proceed to add more covariates; otherwise, we would plan to choose the two covariates that appear to hold the highest unique correlations with IV and DVs.

Analyses will be conducted using two-tailed tests with alpha set to .05.

Our core hypotheses will be tested using two parallel structural equation models (SEM) to consider mania and depression separately. In each, we will examine paths from TRE adherence levels to a latent variable for clock gene amplitude (based on 3 genes) to symptoms to QoL, controlling expected covariates and evaluating the potential confound of glucose tolerance mediation.

We have planned analyses based on availability of data from 125 participants, allowing for attrition of 25 participants. We assume moderate effects for diurnal amplitude on symptoms and QoL (f2 = .25) because smaller effects are inconsistent with the circadian rhythm theory. Assuming f2 = .40 (symptoms to QoL), power to detect the indirect effect of change in amplitude of gene expression on QoL via symptom change is .81, and for full model (RMSEA) > .90 [130, 131]. Intervention-related effects will be estimated using an intention-to-treat approach, in which we will examine final scores (where available) for all participants.

#### Secondary analyses

Within analyses for H4, the potential moderation effects of age of onset, we will consider the effects of age of onset as an interaction term with TRE adherence in the above-described SEM models. For TRE-related changes in secondary outcomes, we will use SEM models paralleling those used for primary hypotheses to determine TRE changes in sleep (actigraphy), emotional variability (assessed by EMA) at 8 weeks compared to baseline.

## Discussion

This study will provide novel evidence about the mechanisms of change for TRE in BD. Previous work suggests that TRE has potent influences on circadian function, allowing a test of whether changes in the diurnal amplitude of clock gene expression help predict improvements in symptoms and downstream QoL. Although we focus on strong hypotheses regarding diurnal amplitude of clock gene expression, we will gather a rich dataset of diurnal parameters, which will allow exploratory analyses of which aspects of diurnal function are most clearly changed by TRE, and which of those changes are most critically relevant for symptom improvement. At the same time, we will consider the extent to which TRE changes other key biological parameters associated with BD outcomes—namely, pro-inflammatory cytokines as markers of inflammation. Taken together, we will assess the benefits of TRE for three critically important processes tied to BD.

Understanding more about the mechanisms of change could help consider a broader array of medical as well as psychiatric outcomes that would be suitable targets of TRE. Given the high prevalence of comorbid medical conditions and their prospective links to poorer psychiatric outcomes, enriching the toolkit for addressing these key biological processes is an important goal. The rich information about possible mechanisms will also help refine understanding of which individuals might be most likely to benefit from TRE. Assessing a range of outcomes and mechanisms related to TRE in BD has important public health implications, as TRE can be easily disseminated worldwide using online technologies.

Despite the novel strengths of the current study, there are important limitations to acknowledge. Although we are building on intervention tools that have been used successfully in medical populations, it is possible that more intensive support and coaching will be needed for those with BD to successfully implement these lifestyle changes. Hence, future research may need to refine how TRE is provided for those with BD. Second, there are significant weaknesses in our assessments of diurnal function. Although we have taken a multi-modal, rich approach to assessing many diurnal parameters, true circadian functions cannot be effectively assessed without a constant routine paradigm [132]. We see the current study as a first step toward the goal of understanding which expressions of circadian function might be shifted with TRE, thereby opening the door for more careful mechanistic work in future studies.

## Data Availability

After the data collection has been completed, de-identified data, data analysis scripts, statistical analysis plan, informed consent, publicly available (non-copyrighted) measures will be shared on the Open Science Foundation within one year after data collection ends, where it will be available upon request. We will also comply with Wellcome Trust guidance for data sharing.

## Abbreviations

AUDIT: Alcohol use disorder identification test
BD: Bipolar disorder
BSRS: Brief Social Rhythm Scale
CGI: Clinical global impression
CMP: Comprehensive metabolic panel
Core CSD: Core consensus sleep diary
C-SSRS: Columbia suicide severity rating scale
DIAMOND: Diagnostic interview for anxiety, mood, and ocd and related neuropsychiatric disorders
DLMO: Dim light melatonin onset
DUDIT: Drug use disorder identification test
EDE-QS: Short eating disorder examination questionnaire
EMA: Ecological momentary assessment
GAD-7: General anxiety disorder
HFSSM: Household food security survey
ICF: Informed consent form
IPSRT: Interpersonal and social rhythm therapy (IPSRT)
IV: Intradaily variability
IS: Interdaily stability
ISI: Insomnia severity index
LIFE: Longitudinal interval follow-up evaluation
MADRS: Montgomery Asberg depression scale
MCTQ: Munich chronotype questionnaire
MENQoL: Menopause QoL items on sleep disruption
MZ: Mood zoom
OCD: Obsessive compulsive disorder
OMC: Orientation memory concentration test
PGI: Patient global impression
PHQ-9: Patient health questionnaire
PROMIS: Patient-reported outcomes measurement information system
QoL.BD: Quality of life in bipolar disorder
RMT20: Rapid measurement toolkit-20
SCISD-R: Structured clinical interview for sleep disorders
SCN: Suprachiasmatic nucleus
SCRSD-R: Structured clinical interview for sleep disorders-revised
TRE: Time-restricted eating
TRF: Time-restricted feeding
WHODAS: World health organization disability assessment schedule
YMRS: Young mania rating scale
